# The transition state structure for binding between TAZ1 of CBP and the disordered Hif-1α CAD

**DOI:** 10.1038/s41598-018-26213-x

**Published:** 2018-05-18

**Authors:** Ida Lindström, Eva Andersson, Jakob Dogan

**Affiliations:** 1 Department of Biochemistry and Biophysics, Stockholm University, 10691 Stockholm, Sweden; 2 Department of Medical Biochemistry and Microbiology, Uppsala University, 75123 Uppsala, Sweden

## Abstract

Intrinsically disordered proteins (IDPs) are common in eukaryotes. However, relatively few experimental studies have addressed the nature of the rate-limiting transition state for the coupled binding and folding reactions involving IDPs. By using site-directed mutagenesis in combination with kinetics measurements we have here characterized the transition state for binding between the globular TAZ1 domain of CREB binding protein and the intrinsically disordered C-terminal activation domain of Hif-1α (Hif-1α CAD). A total of 17 Hif-1α CAD point-mutations were generated and a Φ-value binding analysis was carried out. We found that native hydrophobic binding interactions are not formed at the transition state. We also investigated the effect the biologically important Hif-1α CAD Asn-803 hydroxylation has on the binding kinetics, and found that the whole destabilization effect due the hydroxylation is within the dissociation rate constant. Thus, the rate-limiting transition state is “disordered-like”, with native hydrophobic binding contacts being formed cooperatively after the rate-limiting barrier, which is clearly shown by linear free energy relationships. The same behavior was observed in a previously characterized TAZ1/IDP interaction, which may suggest common features for the rate-limiting transition state for TAZ1/IDP interactions.

## Introduction

It has been recognized for some time that intrinsically disordered proteins (IDPs) and regions (IDRs), constitute a substantial fraction of the eukaryotic proteome^1–5^. Their frequent involvement in various biological processes have during the last decade sparked a significant interest on the role of disorder for protein function^6,7^. Most of these studies have been performed on IDPs and their complexes under equilibrium conditions, and while such studies are crucial for a better understanding of the relationship between disorder and function, experimental kinetic studies are imperative in order to delineate the binding mechanisms and the nature of the transition state for binding, but unfortunately such studies are much less prevalent.

**Figure 1:**
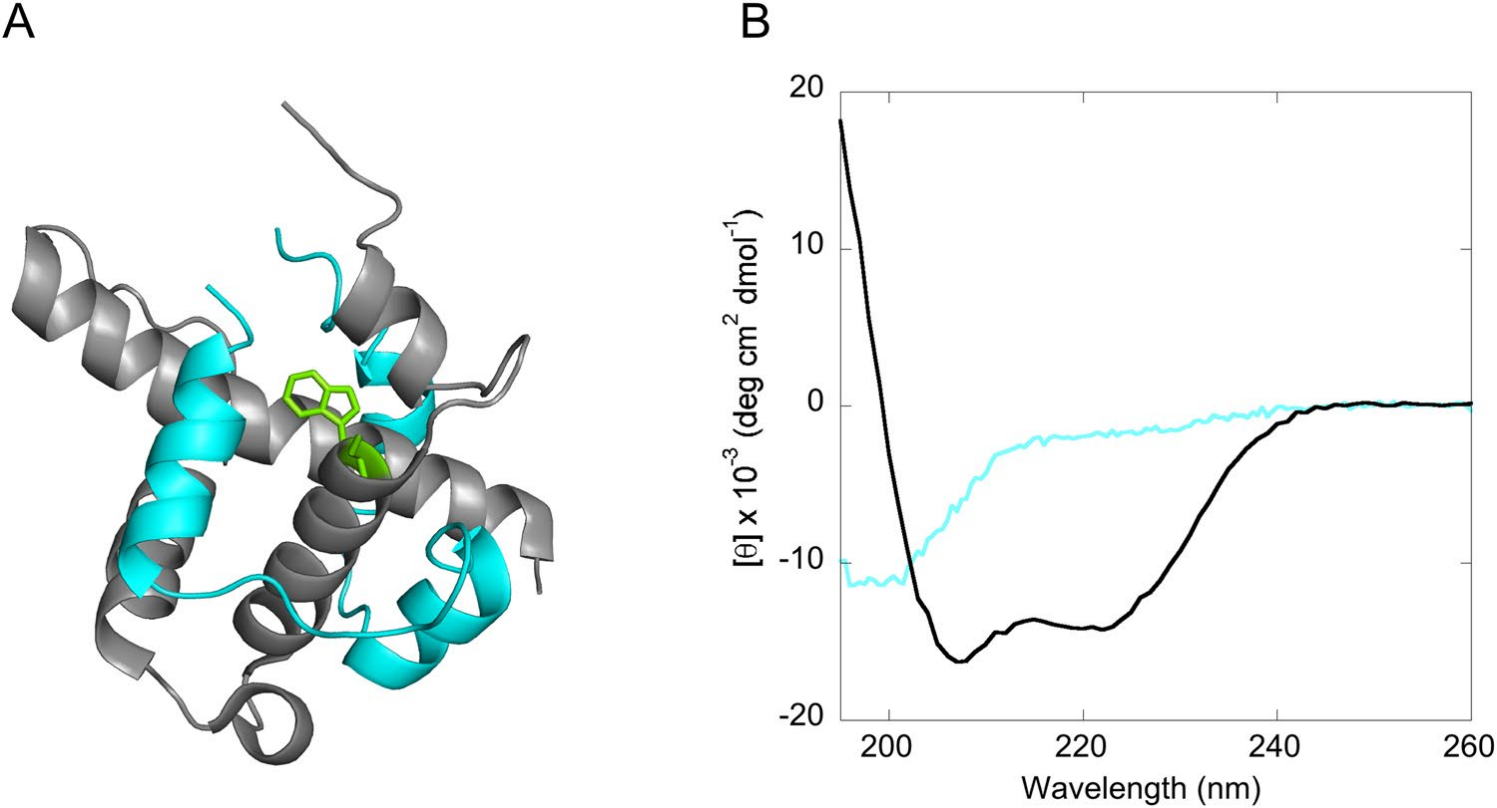
(**A**) 3D structure of TAZ1/Hif-1α CAD (pdb code 1L8C). The backbone of TAZ1 is shown in gray and for Hif-1α CAD it is shown in cyan. The tryptophan in TAZ1, shown here in green is used in this study as a fluorescence probe. (**B**) CD spectrum of Hif-1α CAD (cyan), and TAZ1 (gray). The CD for TAZ1 was previously reported by Lindström and Dogan^12^.

The transcriptional adaptor zinc-binding 1 (TAZ1) is a protein domain of CREB binding protein (CBP) that serves as a recognition domain for a multitude of targets, and plays an important role in transcriptional regulation^3^. Many of its binding partners are IDPs^3^, including the C-terminal activation domain of hypoxia inducible factor 1α (Hif-1α CAD)^8^. The TAZ1/Hif-1α CAD interaction has a significant role in the transcriptional regulation of genes that are crucial for cell survival during low levels of oxygen^9^. During normoxic conditions hydroxylation of two prolines in Hif-1α leads to degradation and hydroxylation of Asn-803 in Hif-1α CAD by the hydrolase factor-inhibiting Hif-1 (FIH-1)^10,11^ significantly lowers the affinity of binding to TAZ1. However, during hypoxia, hydroxylation does not occur resulting in protection from degradation and in low nanomolar binding affinity to TAZ1, which leads to the activation of genes that are important for cell survival during hypoxia. The CITED2 protein plays an important role in the negative feedback regulation of Hif-1α in cellular response during hypoxia^11^. The NMR 3D structure of the TAZ1/Hif-1α CAD complex has previously been determined^8^, which shows that Hif-1α CAD wraps around TAZ1 resulting in the formation of three helices (Fig. 1A).

Here, we have used site-directed mutagenesis together with kinetic measurements in order to get a better understanding of the coupled binding and folding reaction between TAZ1 and Hif-1α CAD. We show that native hydrophobic binding interactions at the rate-limiting barrier are largely absent; instead they are formed cooperatively after the transition state. Similar results were obtained in the previously characterized TAZ1/TAD-STAT2 interaction^12^, and may suggest a potential common mechanism for interactions involving TAZ1 and IDPs.

## Results

The aim of the present study was to investigate the structure of the rate-limiting transition state for binding between the globular TAZ1 domain of CBP and the disordered Hif-1α CAD in terms of native binding interactions. The CD spectrum of Hif-1α CAD displays the typical characteristics for a disordered protein (Fig. 1B), which agrees well with the appearance of the ^15^N-heteronuclear single quantum coherence NMR spectrum, which displayed a low degree of peak dispersion^8^. TAZ1 is a well-defined globular protein domain as previously described^13^. We have here generated 17 Hif-1α CAD point-mutant variants, and they are well distributed over the whole protein sequence. These hydrophobic deletion mutations were made at the binding interface. In addition, the biologically important Asn-803 hydroxylation was generated in this work in order to analyze its effect on the binding kinetics.

### Binding kinetics of TAZ1/Hif-1α CAD

**Figure 2:**
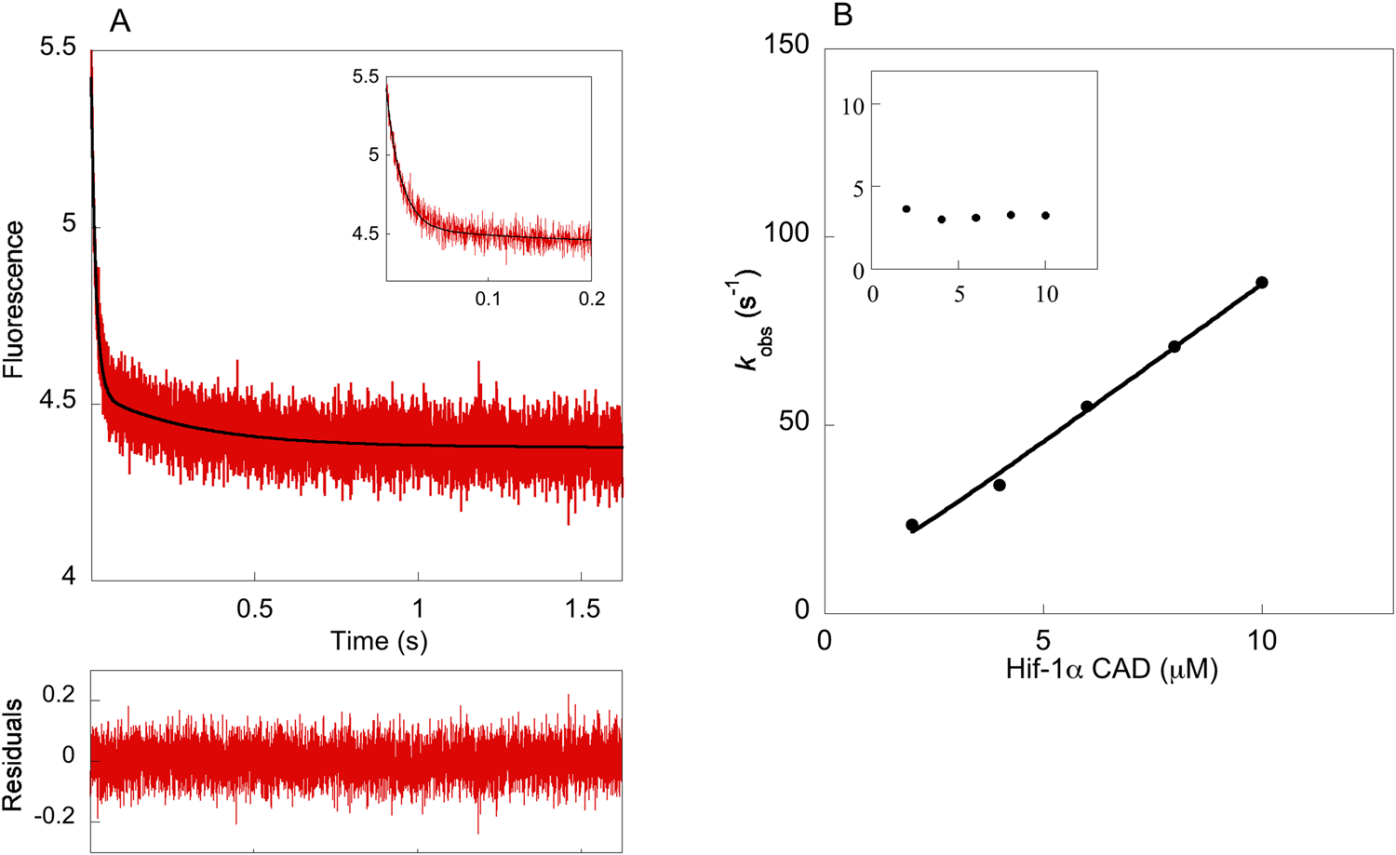
Stopped-flow kinetics for the binding between TAZ1 and Hif-1α CAD. (**A**) Example of a binding kinetics trace for TAZ1/Hif-1α CAD (0.5 μM/8 μM), and a double-exponential function was used for fitting. The inset shows a zoomed view of the fast phase. (**B**) The concentration dependence of *k*_obs_ for the slow (inset) and fast phase. The *k*_obs_ for the fast phase is linearly dependent on Hif-1α CAD concentration while the *k*_obs_ for the slow phase is concentration independent, with a value of 3–4 s^−1^ for all Hif-1α CAD variants. *k*_on_^app^ was obtained by fitting the concentration dependence of *k*_obs_ for the fast phase to the general equation for association of two molecules^28^.

The association kinetics were measured by the stopped-flow technique. The concentration of Hif-1α CAD was varied and the fluorescence change of Trp-418 in TAZ1 was monitored. The association kinetics contained two phases (Fig. 2); a fast phase for which the observed rate constant, *k*_obs_, increased linearly with the concentration of Hif-1α CAD, and a slow phase for which the *k*_obs_ value was not dependent on the concentration, centering at around 3–4 s^−1^. The apparent association rate constant, *k*_on_^app^, was obtained from the linear dependence of Hif-1α CAD concentration of *k*_obs_ for the fast phase, and was found to be equal to 8.8 × 10^6^ M^−1^ s^−1^. The apparent dissociation rate constant, *k*_off_^app^, was obtained from displacement experiments and determined to be 0.030 s^−1^, resulting in a *K*_d_ = *k*_off_^app^/*k*_on_^app^ = 3.4 nM. Biphasic kinetics was also observed in a previous study where the binding between TAZ1 and another IDP, namely TAD-STAT2, was characterized by protein engineering and stopped-flow kinetics^12^. The *k*_obs_ value for the slow phase for TAZ1/TAD-STAT2 is the same as the one obtained for TAZ1/Hif-1α CAD, and as was also the case for TAZ1/TAD-STAT2, this value is very similar among the Hif-1α CAD mutant variants (Supplementary Table S1), which implies that this phase could be a result of a conformational change in TAZ1 that occurs before binding.

### Impact of mutations in Hif-1α CAD to the binding of TAZ1

**Figure 3:**
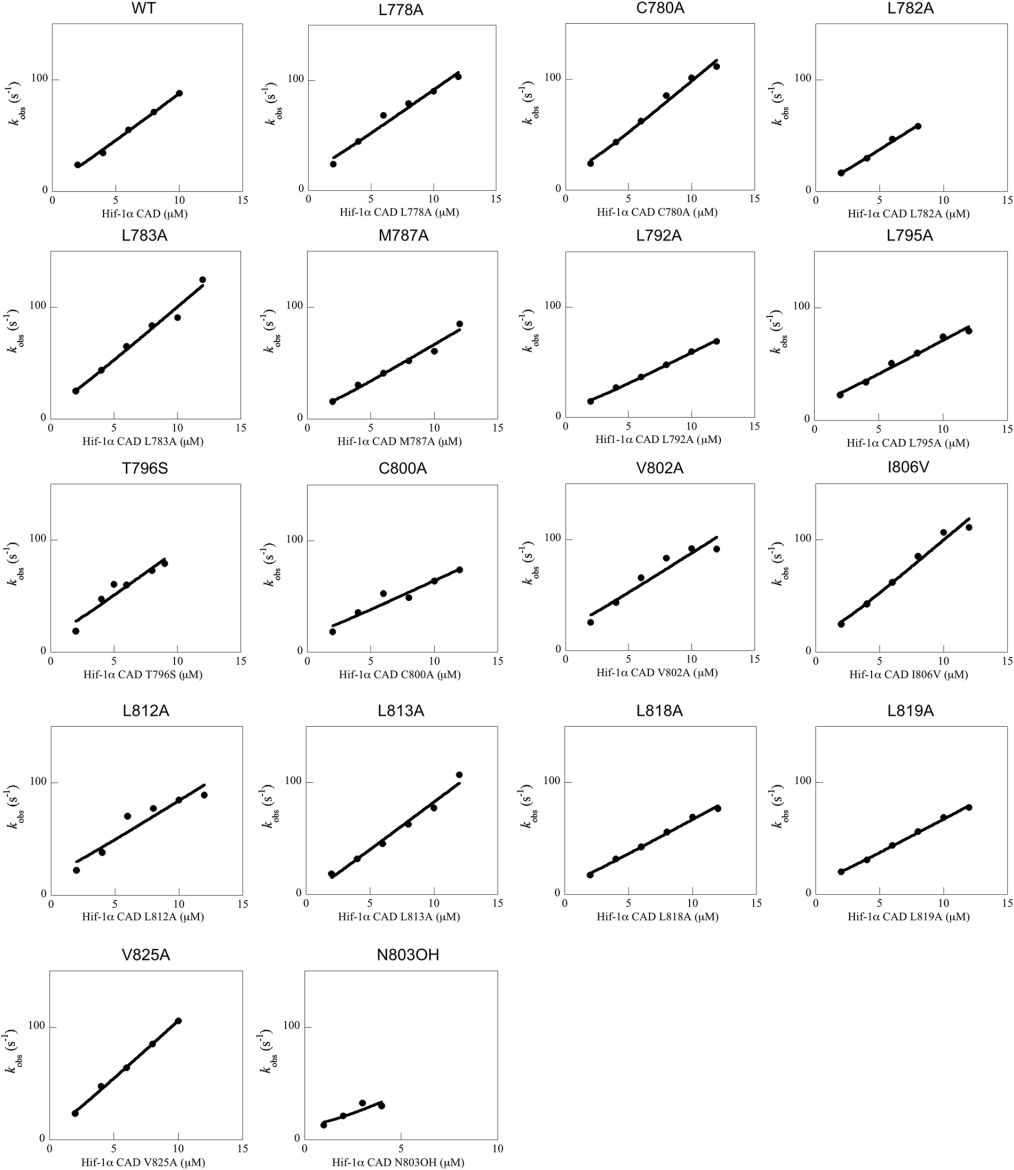
The concentration dependence of *k*_obs_ for the fast phase for all TAZ1/Hif-1α CAD variants. The data was fitted to the general equation for the reversible association of two molecules^28^ in order to obtain *k*_on_^app^.

The binding kinetics for the Hif-1α CAD mutant variants were measured using stopped-flow fluorimetry (Fig. 3 and Supplementary Fig. S1). Point mutations that were made at the N-terminal part of Hif-1α CAD (region 778–787), did not affect the binding affinity appreciably, whereas a significant response was observed for point mutations that were made at region 792–825, with binding affinities being reduced by as much as 300-fold (Table 1). The mutations that destabilized the complex the most (>2 kcal mol^−1^) were L792A, L795A, L813A, and L818A. The Asn-803 hydroxylation lowered the binding affinity by 50-fold. The L818 position is also part of an LLXXL recognition motif in helix 3, where X may represent any amino acid. These motifs are frequently observed in protein-protein associations that play a role in transcriptional regulation^14,15^.

**Table 1:** Binding parameters for TAZ1/Hif-1α CAD variants.

Hif-1α CAD variant	*k*_on_^app^ μM^−1^ s^−1^	*k*_off_^app^ s^−1^	*K*_d_ nM	ΔΔ*G*_Eq_ kcal mol^-1^	ΔΔ*G*_TS_ kcal mol^-1^	Φ
WT	8.83 ± 0.35	0.0297 ± 0.0003	3.36 ± 0.14			
L778A^a^	8.06 ± 0.68	0.0294 ± 0.0004	3.65 ± 0.31	0.05 ± 0.06	0.05 ± 0.05	—
C780A^a^	9.56 ± 0.50	0.0361 ± 0.0006	3.78 ± 0.21	0.07 ± 0.04	−0.05 ± 0.04	—
L782A^a^	7.36 ± 0.34	0.0336 ± 0.0003	4.56 ± 0.21	0.18 ± 0.04	0.11 ± 0.03	—
L783A^a^	9.62 ± 0.63	0.0346 ± 0.00004	3.60 ± 0.24	0.04 ± 0.04	−0.05 ± 0.04	—
M787A^a^	6.70 ± 0.45	0.0324 ± 0.0015	4.83 ± 0.39	0.21 ± 0.05	0.16 ± 0.04	—
L792A	5.75 ± 0.14	0.920 ± 0.195	160 ± 34	2.25 ± 0.13	0.25 ± 0.03	0.11 ± 0.01
L795A	6.12 ± 0.38	6.74 ± 0.20	1101 ± 76	3.37 ± 0.05	0.21 ± 0.04	0.06 ± 0.01
T796S^a^	8.27 ± 1.24	0.0267 ± 0.0005	3.23 ± 0.49	−0.02 ± 0.09	0.04 ± 0.09	—
C800A	6.55 ± 1.12	0.116 ± 0.002	17.7 ± 3.1	0.97 ± 0.10	0.17 ± 0.10	0.18 ± 0.11
V802A	7.28 ± 1.06	0.0995 ± 0.0019	13.7 ± 2.0	0.82 ± 0.09	0.11 ± 0.09	0.14 ± 0.11
I806V	9.73 ± 0.66	0.0577 ± 0.0019	5.93 ± 0.45	0.33 ± 0.05	−0.06 ± 0.05	−0.17 ± 0.14
L812A	7.12 ± 1.21	0.509 ± 0.0057	71.5 ± 12.1	1.78 ± 0.10	0.13 ± 0.10	0.07 ± 0.06
L813A	8.52 ± 0.53	1.17 ± 0.01	137 ± 9	2.16 ± 0.04	0.02 ± 0.04	0.01 ± 0.02
L818A	6.23 ± 0.23	1.27 ± 0.14	205 ± 24	2.39 ± 0.07	0.20 ± 0.03	0.09 ± 0.01
L819A	6.10 ± 0.15	0.141 ± 0.001	23.1 ± 0.6	1.12 ± 0.03	0.22 ± 0.03	0.19 ± 0.02
L822A^b^						
V825A	10.4 ± 0.3	0.0220 ± 0.0007	2.12 ± 0.09	−0.27 ± 0.03	−0.09 ± 0.03	0.34 ± 0.11
Hif-1α OH	7.7 ± 2.9	1.317 ± 0.004	172 ± 64	2.29 ± 0.22	0.08 ± 0.22	0.04 ± 0.10

^a^A Φ-binding value was not determined since ΔΔ*G*_Eq_ was too low. ^b^This mutant variant did not give reliable kinetic data. The reported error in *k*_off_^app^ is the standard deviation (s.d.) of *k*_obs_ values determined at varying concentrations of TAZ1^W418Y^ in the displacement experiments. The reported uncertainty for *k*_on_^app^ is the error from the fit or s.d. from repeated measurement of *k*_on_^app^.

### Φ-Value binding analysis and structure of the rate-limiting transition state

A Φ-value binding analysis^16^ was carried out in order to determine to what extent native binding contacts are present at the rate-limiting transition state for binding between TAZ1 and Hif-1α CAD. Φ-values are calculated as the ratio between the free energy change for the rate-limiting transition state for binding, ΔΔ*G*_TS_, and at equilibrium, ΔΔ*G*_Eq_:

1 $${\rm{\Delta }}{\rm{\Delta }}{G}_{TS}=RT\,{\rm{l}}{\rm{n}}(\frac{{k}_{on}^{wild{\textstyle  \mbox{-} }type}}{{k}_{on}^{mutant}})$$

2 $${\rm{\Delta }}{\rm{\Delta }}{G}_{Eq}=RT\,{\rm{l}}{\rm{n}}(\frac{{K}_{d}^{mutant}}{{K}_{d}^{wild{\textstyle  \mbox{-} }type}})$$

3 $${\rm{\Phi }}=\frac{{\rm{\Delta }}{\rm{\Delta }}{G}_{TS}}{{\rm{\Delta }}{\rm{\Delta }}{G}_{Eq}}$$

The *K*_d_ values that were used in this analysis were determined as *k*_off_/*k*_on_. We compared the *K*_d_ obtained from the stopped-flow method with that determined by ITC for the Hif-1α CAD L813A mutation (Supplementary Fig. S2), which lowered the binding affinity to such an extent that an accurate determination of *K*_d_ by ITC was possible, and the results show that they are in excellent agreement with each other (*K*_d_ by the stopped-flow technique: 0.137 ± 0.009 μM, and *K*_d_ by ITC: 0.144 ± 0.012 μM). A Φ-binding value of zero means that the residue that has been mutated does not form native contacts at the transition state for binding, while a Φ–binding value of 1 means that the change in *K*_d_ is the same as the change in *k*_on_, thus the native interaction is completely present at the rate-limiting transition state for binding. Thus, the Φ-binding value is an index with values between 0 and 1 being interpreted as partially formed native binding contacts at the rate-limiting transition state for binding. A Φ-value was determined if the magnitude of ΔΔ*G*_Eq_ was larger or the same as 0.3 kcal mol^−1^. Two mutations (I806V and V825A) had a magnitude value of ΔΔ*G*_Eq_ that was equal to 0.3 kcal mol^−1^, whereas the rest of the calculated Φ-values had an associated ΔΔ*G*_Eq_ that was larger than 0.8 kcal mol^−1^ (Table 1). Φ-values for the I806V and V825A mutations were calculated due to the high precision in the obtained rate constants.

**Figure 4:**
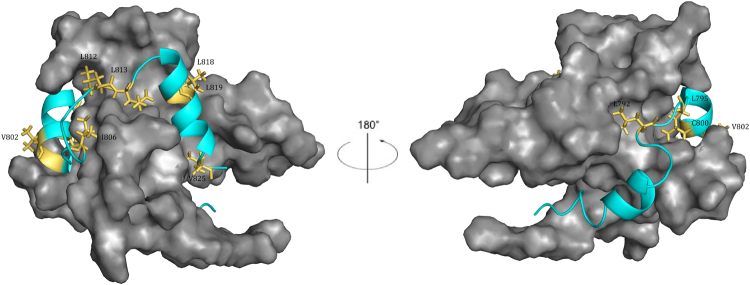
All positions for which we were able to determine a Φ-binding value are highlighted in yellow, and are labeled in the figure. The backbone of Hif-1α CAD is shown in cyan, and the surface of TAZ1 is shown in gray. All Φ–binding values were low (≤0.34). Images were produced with PyMol.

**Figure 5:**
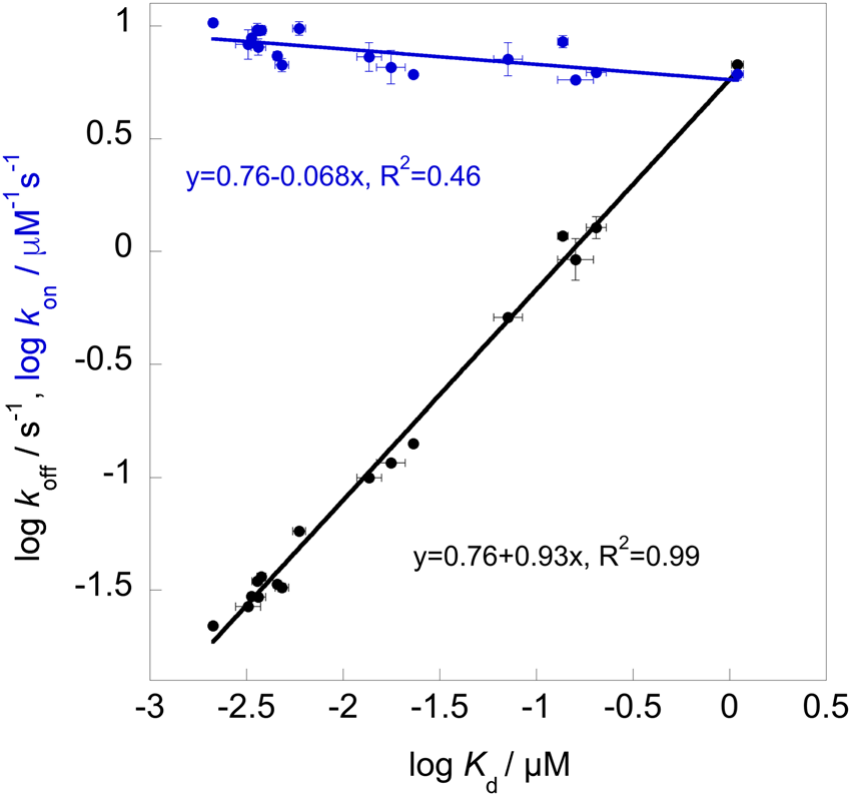
Linear free energy relationships. log *k*_off_^app^ vs log *K*_d_ is shown in black, while log *k*_on_^app^ vs log *K*_d_ is shown in blue. Each point in the graph represents a TAZ1/Hif-1α CAD variant (Table 1).

All of the determined Φ-binding values are low (≤0.34) which demonstrates that native hydrophobic binding interactions have not been created yet at the rate-limiting transition state for binding between TAZ1 and Hif-1α CAD (Table 1 and Fig. 4), suggesting that the rate-limiting transition state is disordered-like with low presence of native contacts in the binding interface. The whole effect of the hydroxylation of Asn-803 in Hif-1α CAD is within the dissociation rate constant (Table 1), which in turn results in a Φ-value that is low. This demonstrates that while the association kinetics for this hydroxylation remains about the same as the wild-type, the introduction of an OH-group significantly disturbs the interaction between TAZ1 and Hif-1α CAD-OH, resulting in a large increase in *k*_off_. Linear free energy relationships^17,18^ (LFER) (Fig. 5) clearly demonstrate that the destabilizing effect upon mutation is dominated by changes in the dissociation rate constant. As shown in Fig. 5, the linearity and a slope that is close to 1 for log *k*_off_^app^ vs log *K*_d_, means that native non-polar binding interactions are cooperatively created after the rate-liming barrier, which was also observed to be the case for the binding of TAZ1 to TAD-STAT2^12^, suggesting that TAZ1/IDP interactions may share similar transition state features. However, further studies on other TAZ1/IDP binding reactions need to be performed to see if this is the case.

## Discussion

The use of protein engineering in combination with kinetics measurements have during the last couple of years started to give us a better understanding on the binding mechanisms and short-lived intermediates involving binding of intrinsically disordered proteins. However, it remains unclear regarding the generality of certain mechanisms, for instance if the binding proceeds through a rate-limiting barrier that resembles the ground state structure in terms of native contact formation or not^18,19^. Such studies are also useful for benchmarking MD simulations. The analysis of the binding kinetics for TAZ1/Hif-1α CAD mutant variants reveal that native hydrophobic binding contact formations at the rate-limiting transition state are absent, since all of the calculated Φ-values are low. This was also shown to be the case in a previous study where a similar analysis was performed for the interaction between TAZ1 and the disordered TAD-STAT2^12^. Even though the topology of both Hif-1α CAD^8^ and TAD-STAT2^20^, which wrap around TAZ1, is rather different compared to the single α-helix conformation that represents the bound IDP in other binding reactions^17,21,22^, the end-result of these studies are, with the exception of a few studies^22^, quite similar, i.e. the rate-limiting transition state for binding is rather unstructured, in agreement with MD simulations studies^23,24^. For instance, low Φ-binding values were consistently obtained for the interaction between PDZ domain and peptide targets^17^, and in S-protein/S-peptide interactions^25^. Similar results were also reported by Rogers *et al*.^21^, which showed that the transition state in the interaction between PUMA (an IDP that adopts a single α–helix when bound) and the globular MCL-1 is rather unstructured, as was also the case for the interaction between the molten-globule-like NCBD and the disordered activation domain of ACTR^26^. Although, there are a few examples where high Φ-binding values have been determined for IDP binding interactions^22^, generally it seems that the rate-limiting transition state is lacking to a large extent native hydrophobic binding contacts, forming late on the reaction pathway. Furthermore, the linearity in the LFER plot with a slope of 0.93 for log *k*_off_^app^ vs log *K*_d_ clearly show that native hydrophobic interactions in TAZ1/Hif-1α CAD are created cooperatively after crossing the rate-limiting transition state, which was also observed for TAZ1/TAD-STAT2^12^ and other systems as well^17,21,26^, suggesting that these features might be common in IDP binding reactions. Studies on other IDP associations need however to be performed in order to find out if there is a general trend.

It has usually been observed that certain positions contribute more to the binding energetics than other interacting residues in protein-ligand associations. A common way of defining these so-called hot-spots, is that a mutation to Ala reduces the binding affinity by more than 2 kcal mol^−1^ ^27^. An intriguing result from the present study is that the largest mutational destabilizations of the TAZ1/Hif-1α CAD interaction are only observed to take place for substitutions at the 792–825 region in Hif-1α CAD, thus none of the mutations at the N-terminal part 778–787 resulted in significant changes (Table 1). Positions L792, L795, L813, and L818 were identified as hot-spots, which also includes a position (L818) within an LLXXL recognition motif, which are known to be important in many protein-protein associations^14^. Thus, as reported in Table 1, the very low presence of hydrophobic native binding interactions at the transition state involve all of these hot-spots as well.

In conclusion, we have shown that the rate-limiting transition state structure does not contain native hydrophobic binding interactions, but form cooperatively after crossing the rate-limiting barrier for binding between TAZ1 and Hif-1α CAD.

## Materials and Methods

### Protein expression and purification

Human TAZ1 (residues 340−439) was expressed and purified as described previously^12^. Hif-1α CAD (residues 776–826) was cloned into a pRSET vector, directly downstream of a His_6_-lipoyl domain−thrombin site tag. FIH-1 was inserted into the pET28a(+) vector, with a N-terminal His_6_-thrombin tag preceding the FIH-1 sequence. Variants of the Hif-1α CAD were obtained using the quick exchange approach with pfu Ultra and DpnI digestion. Plasmids were propagated using XL1 blue cells (Agilent) and the sequences were verified by DNA sequencing (Eurofins). Hif-1α CAD and FIH-1 were transformed into *E. coli* BL21 pLysS cells (Thermo Fisher Scientific). The cells were cultivated in 2xTY medium at 37 °C to an OD_600_ of 0.6–0.7, followed by induction with 0.8 mM isopropyl β-D thiogalactopyranoside. The cells were grown further for 16–18 hours at 18 °C for Hif-1α CAD and 15 °C for FIH-1. Cell lysis was performed by sonication, followed by centrifugation in order to remove the cell debris, after which the supernatant was passed through a 0.2 μm filter and subsequently added to a prequilibrated nickel affinity column. The column was washed with buffer (20 mM Tris-HCl (pH = 8.0), 1 mM TCEP, 500 mM NaCl, and 5 mM imidazole) after which protein elution was done with 20 mM Tris (pH = 7.9), 1 mM TCEP, 250 mM imidazole, 500 mM NaCl. The protein solution was dialyzed overnight at 4 °C against 20 mM Tris (pH = 8.0), 1 mM TCEP, and 100 mM NaCl. Thrombin was added to the protein solution, and left to cleave for 6–8 hours at room temperature. The cleaved protein was loaded onto the sepharose nickel column to separate the fusion-tag and other impurities from the cleaved protein, which was present in the flow through. FIH-1 was dialyzed into a buffer of 20 mM Tris (pH = 7.5), 150 mM NaCl and 0.5 mM DTT overnight and then further used for hydroxylation of Hif-1α CAD. As a final purification step for Hif-1α CAD, reversed phase (RP) chromatography was used with a Resource RP column (GE Healthcare) where water/acetonitrile solvents with 0.1% (v/v) trifluoroacetic acid were used. The identity of all Hif-1α CAD variants were verified with matrix-assisted laser desorption ionization time-of-flight (MALDI-TOF) mass spectrometry.

### FIH-1 mediated hydroxylation

For the hydroxylation of Hif-1α CAD, the purification of Hif-1α CAD was halted after the second sepharose nickel column step and was instead dialyzed into a buffer containing 20 mM Tris (pH = 7.2), 1 mM DTT and 150 mM NaCl. Thereafter, FIH-1 was added in a 1:10 (FIH-1/Hif-1α CAD) molar ratio containing 20 mM Tris (pH = 7.4), 150 mM NaCl, 1 mM DTT, 4 mM ascorbic acid, 1.5 mM FeSO_4_, 1 mM MgCl_2_, 40 µM 2-oxoglutarate and 1 mM PMSF. The hydroxylation was conducted at 37 °C for four hours. Thereafter the purification of Hif-1α CAD was continued as described above with reversed phase chromatography. The hydroxylation was confirmed with MALDI-TOF mass spectrometry.

### Stopped-flow fluorimetry

Stopped-flow fluorimetry experiments were taken using a SX-18MV stopped-flow spectrometer (Applied Photophysics, Leatherhead, U.K.) at 293 K. TAZ1 contains a single tryptophan which was utilized as the fluorescence probe. Excitation was done at 295 nm and a 320 nm long pass-filter was used when monitoring the fluorescence change. Samples were prepared in 20 mM HEPES (pH = 6.9), 1 mM TCEP, and 190 mM NaCl. For measurement of the association rate constants the TAZ1 concentration was kept constant at 0.5–0.9 *µ*M and the concentration of Hif-1α CAD and its variants were varied between 1 *µ*M and 15 *µ*M. The binding traces were biphasic for both the wild type and all of the variants of Hif-1α CAD, with a slow phase and a fast phase. The observed rate constant (*k*_obs_) for the slow phase was concentration independent and similar for both the Hif-1α CAD and its variants (Supplementary Table S1). The *k*_obs_ for the fast phase showed a linear Hif-1α CAD concentration dependence in the range of concentrations at which data were collected. The concentrations used here are mostly at pseudo-first order conditions. By using these *k*_obs_ values, the apparent association rate constant (*k*_on_^app^) was determined by fitting the data to the general equation for the reversible association of two molecules^28^. The dissociation rate constant (*k*_off_^app^) was determined by displacement experiments in which a TAZ1 variant with the tryptophan being replaced by a tyrosine (TAZ1^W418Y^) was utilized^12^. A complex solution of TAZ1/Hif-1α CAD variant (0.75 *µ*M/0.5* µ*M) was mixed with an excess of TAZ1^W418Y^ (varied between 10 *µ*M and 50 *µ*M), in which TAZ1^W418Y^ displaces TAZ1, resulting in traces that were single-exponential. At excess concentrations of TAZ1^W418Y^ the *k*_obs_ is equal to *k*_off_^app^. The dissociation constant, *K*_d_, was then determined as *K*_d_* = k*_off_^app^*/k*_on_^app^.

### Circular dichroism (CD) spectroscopy

CD spectrum in the far-uv region was recorded for Hif-1α CAD using a Chirascan spectrometer using a 1 mm cuvette and at 298 K. The protein concentration was 10 μM and in a buffer containing 5 mM HEPES (pH = 6.9), 1 mM TCEP, and 50 mM NaCl.

### Isothermal titration calorimetry (ITC)

ITC experiments were taken using an iTC200 (Malvern Instruments) calorimeter, at 293 K. The proteins were dialyzed against 20 mM HEPES (pH = 6.9), 1 mM TCEP, and 190 mM NaCl prior to ITC measurements. A 15 *µ*M TAZ1 solution was loaded onto the calorimeter cell, while Hif-1α CAD (L813A) was in the syringe (149 *µ*M). The titration series started with a 1.3 *µ*L injection, followed by 19, 1.8 *µ*L injections. Fitting of the binding isotherm was carried out using a one-to-one model.

## Electronic supplementary material

Supplementary Information
