# Supplementary material for: The transition state structure for binding between TAZ1 of CBP and the disordered Hif-1α CAD
Source: Sci Rep. 2018 May 18;8:7872. doi: 10.1038/s41598-018-26213-x (PMC5959852; doi:10.1038/s41598-018-26213-x)
Supplement: Supplementary file 1 — Supplementary Information [file 41598_2018_26213_MOESM1_ESM.pdf]

Supplementary Information

# The transition state structure for binding between TAZ1 of CBP and the disordered Hif-1α CAD

Ida Lindström[1], Eva Andersson[2], and Jakob Dogan[1*]

[1]Department of Biochemistry and Biophysics, Stockholm University, 10691 Stockholm, Sweden

[2]Department of Medical Biochemistry and Microbiology, Uppsala University, 75123 Uppsala, Sweden

* Corresponding author: e-mail: jakob.dogan@dbb.su.se

Telephone: 46-8-162470

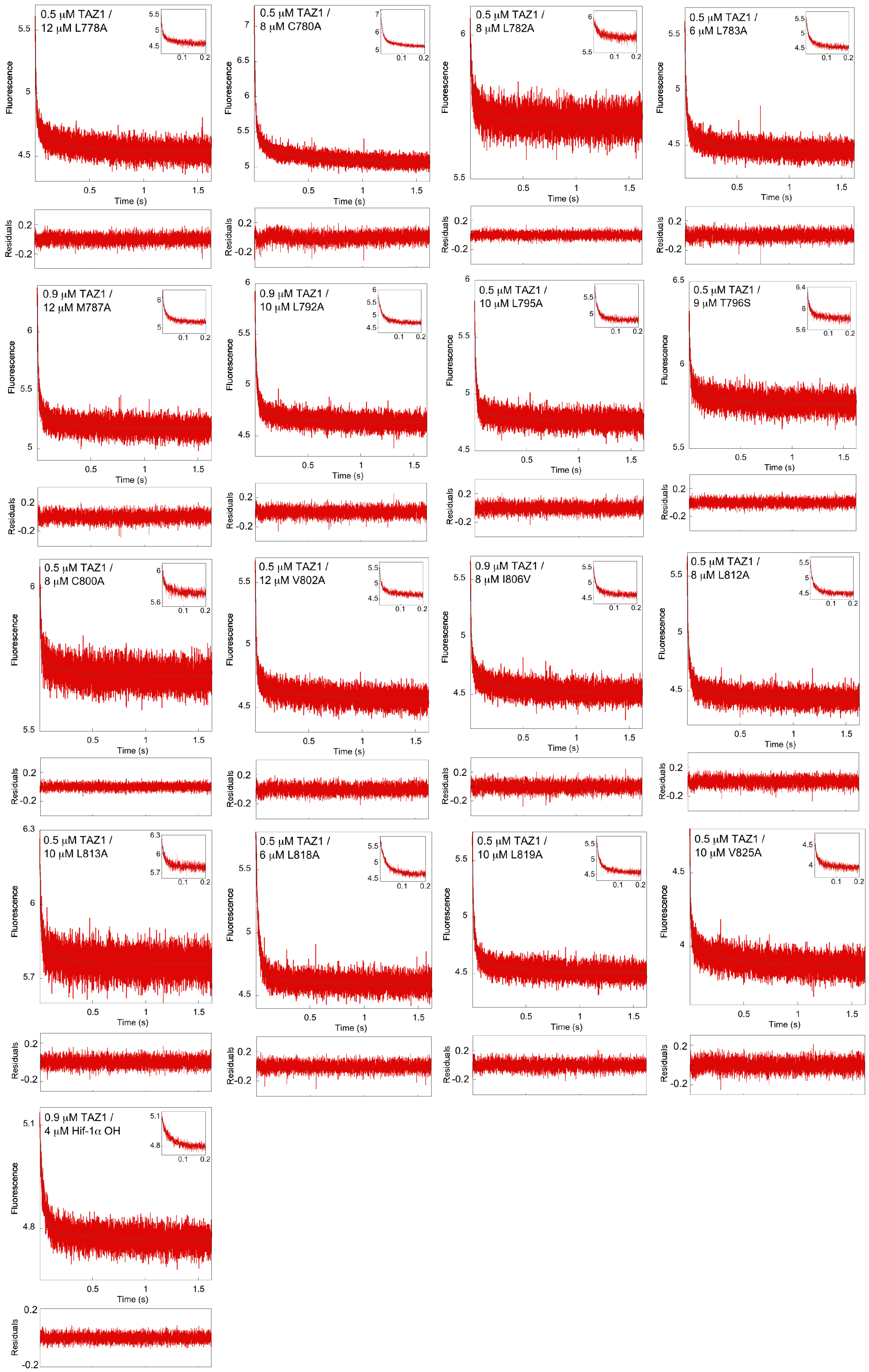


**Figure S1.** Representative stopped-flow binding traces for the TAZ1/Hif-1α CAD variants. The inset shows a closer view of the fast phase. Data were fitted to a double exponential function.

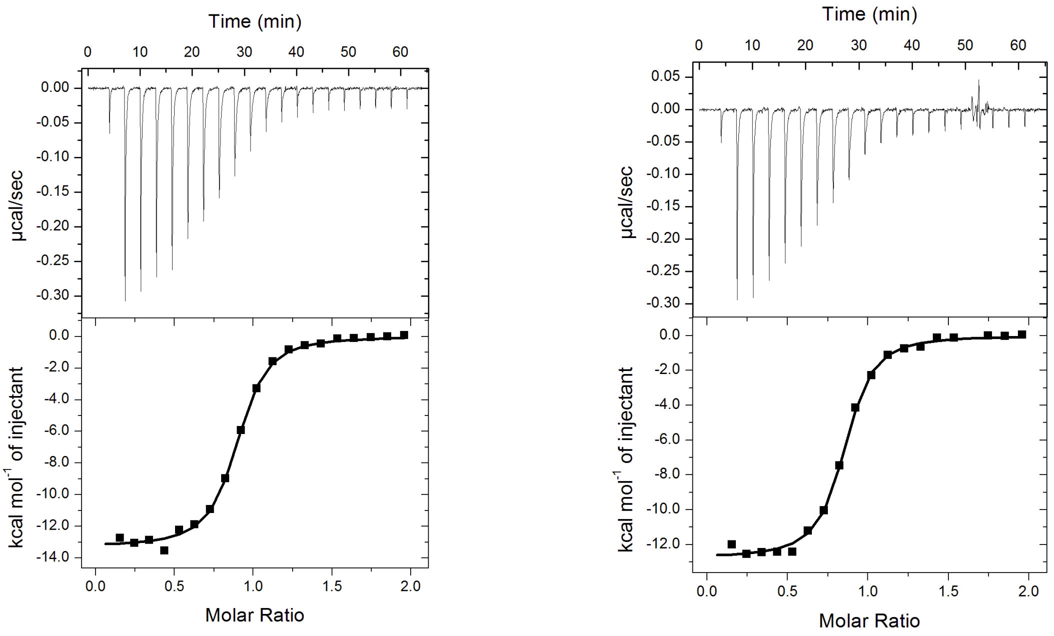


**Figure S2.** ITC titration series for the interaction between TAZ1 and Hif-1α CAD (L813A), which were performed in duplicate, both of which are shown here. The determined average $K_d$ is 0.144 ± 0.012 μM, which is in excellent agreement with the $K_d$ obtained with the stopped-flow method (0.137 ± 0.009 μM).

**Table S1.** $k_{obs}$ for the slow phase for the binding between TAZ1 and Hif1-1α CAD mutant variants.

| **Hif-1α CAD variant** | **$k_{obs}$ ($s^{-1}$) slow phase** | **error** |
|---|---:|---:|
| WT | 3.33 | 0.39 |
| L778A | 3.22 | 0.23 |
| C780A | 3.40 | 0.33 |
| L782A | 3.29 | 0.51 |
| L783A | 3.32 | 0.29 |
| M787A | 3.19 | 0.64 |
| L792A | 2.86 | 0.31 |
| L795A | 2.70 | 0.52 |
| T796S | 3.01 | 0.35 |
| C800A | 3.24 | 1.17 |
| V802A | 3.56 | 0.57 |
| I806V | 3.18 | 0.37 |
| L812A | 3.59 | 0.20 |
| L813A | 4.39 | 1.24 |
| L818A | 3.81 | 0.75 |
| L819A | 3.62 | 0.66 |
| V825A | 2.69 | 0.14 |
| Hif-1α OH | 4.97 | 1.71 |
